# Inhibition of chemerin/CMKLR1 axis in neuroblastoma cells reduces clonogenicity and cell viability *in vitro* and impairs tumor growth *in vivo*

**DOI:** 10.18632/oncotarget.19619

**Published:** 2017-07-27

**Authors:** Conny Tümmler, Igor Snapkov, Malin Wickström, Ugo Moens, Linda Ljungblad, Lotta Helena Maria Elfman, Jan-Olof Winberg, Per Kogner, John Inge Johnsen, Baldur Sveinbjørnsson

**Affiliations:** ^1^ Molecular Inflammation Research Group, Department of Medical Biology, Faculty of Health Science, University of Tromsø, Tromsø, Norway; ^2^ Childhood Cancer Research Unit, Department of Women's and Children's Health, Karolinska Institutet, Stockholm, Sweden; ^3^ Tumor Biology Research Group, Department of Medical Biology, Faculty of Health Science, University of Tromsø, Tromsø, Norway

**Keywords:** pediatric cancer, neuroblastoma, inflammation, GPCR, chemerin

## Abstract

Pro-inflammatory cells, cytokines, and chemokines are essential in promoting a tumor supporting microenvironment. Chemerin is a chemotactic protein and a natural ligand for the receptors CMKLR1, GPR1, and CCRL2. The chemerin/CMKLR1 axis is involved in immunity and inflammation, and it has also been implicated in obesity and cancer.

In neuroblastoma, a childhood tumor of the peripheral nervous system we identified correlations between high *CMKLR1* and *GPR1* expression and reduced overall survival probability. CMKLR1, GPR1, and chemerin RNA and protein were detected in neuroblastoma cell lines and neuroblastoma primary tumor tissue. Chemerin induced calcium mobilization, increased MMP-2 synthesis as well as MAP-kinase- and Akt-mediated signaling in neuroblastoma cells. Stimulation of neuroblastoma cells with serum, TNFα or IL-1β increased chemerin secretion. The small molecule CMKLR1 antagonist α-NETA reduced the clonogenicity and viability of neuroblastoma cell lines indicating the chemerin/CMKLR1 axis as a promoting factor in neuroblastoma tumorigenesis. Furthermore, nude mice carrying neuroblastoma SK-N-AS cells as xenografts showed impaired tumor growth when treated daily with α-NETA from day 1 after tumor cell injection.

This study demonstrates the potential of the chemerin/CMKLR1 axis as a prognostic factor and possible therapeutic target in neuroblastoma.

## INTRODUCTION

Neuroblastoma is a malignancy of the sympathetic nervous system occurring in early childhood and accounting for 7% of all pediatric cancers [1]. While the prognosis for low and intermediate risk neuroblastoma patients is favorable, the long-term event-free survival rate for high-risk patients remains less than 50%, despite intensive treatment [1, 2].

Chronic inflammation is an important modulator of the tumor microenvironment (TME). Pro-inflammatory cells, cytokines, and chemokines present in the TME promote tumor development, progression, and metastasis in various cancers [3, 4]. Recently, a subset of high-risk, therapy-resistant neuroblastomas was demonstrated to be inflammation-driven indicating the importance of inflammation in neuroblastoma [5]. A thorough understanding of the neuroblastoma TME and the inflammatory processes involved in tumorigenesis may lead to new therapy approaches and the discovery of novel prognostic markers [6–9].

Chemerin (also known as TIG-2 or RARRES2) is an adipokine and chemoattractant factor associated with obesity, inflammatory diseases and cancer [10–20]. Synthesized as a 163 amino acid preproprotein, this chemerin precursor is N-terminally cleaved and secreted as prochemerin with low activity. Following secretion, prochemerin can be C-terminally cleaved by a variety of extracellular proteases, resulting in several chemerin isoforms with varying length, receptor affinity, and biological activity [21]. Proteases associated with inflammation such as cathepsin G and neutrophil elastase respectively cleave prochemerin into chemerin 21-156 and 21-157. These are the most active forms, whereas prochemerin processed with mast cell chymase or protease 3 results in the inactive or low activity chemerin 21-154 and 21-155 variants, respectively [22, 23]. During inflammation initiation, maintenance and resolution the different chemerin isoforms may exert pro- and/or anti-inflammatory functions [24, 25]. Chemerin is a natural ligand for the G-protein-coupled receptors CMKLR1 (or ChemR23), GPR1, and CCRL2. CMKLR1 is expressed by different cell types including macrophages as well as immature dendritic cells and mediates the majority of the described chemerin functions [10, 24, 26–29]. Besides the involvement in various inflammatory diseases, the chemerin/CMKLR1 axis has been shown to play a role in different malignancies. While there is evidence that chemerin and CMKLR1 support tumorigenesis in glioblastoma, gastric cancer, squamous esophageal cancer and squamous cell carcinoma of the oral tongue [16–18, 20], an anti-tumorigenic effect has been suggested in melanoma, hepatocellular carcinoma and non-small cell lung cancer [15, 30, 31].

GPR1 functions are so far less understood, but it has recently been found to contribute to the regulation of glucose homeostasis in obese mice [32]. At present, no active signaling has been detected following chemerin binding to CCRL2. However, CCRL2 is known to increase local chemerin concentrations [33] and its expression has been linked to rheumatoid arthritis and liver metastasis in colorectal cancer [34, 35]. The aim of the present study was to investigate the functional significance of chemerin, CMKLR1 and GPR1 in the neuroblastoma microenvironment and assess their potential as prognostic factors and therapeutic targets.

## RESULTS

### High *CMKLR1* and *GPR1* expression predict poor overall survival probability in neuroblastoma

**Figure 1:**
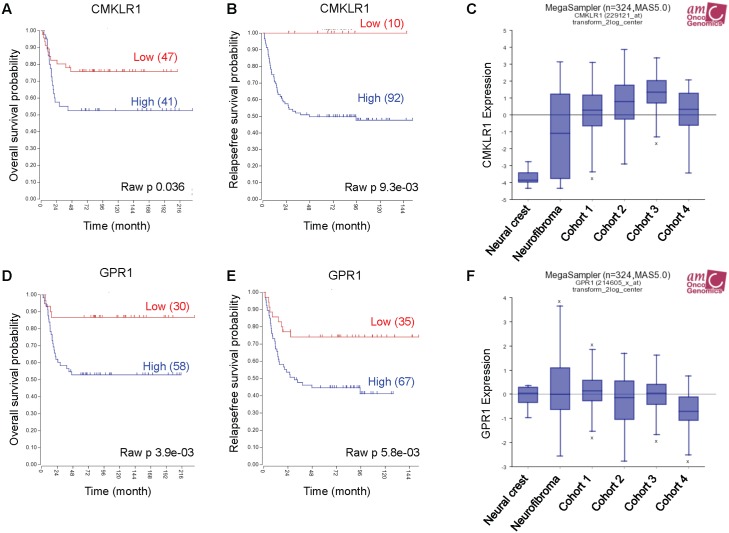
High *CMKLR1* and *GPR1* expression predicts poor survival in neuroblastoma patients Expression data was analyzed using the R2 database http://r2.amc.nl. Kaplan-Meier survival estimates were used to evaluate the prognostic value of *CMKLR1* **(A, B)** and *GPR1* **(D, E)** expression in two patient data sets (A and D: Versteeg n=88; B and E: Seeger n=102). The Kaplan-Meier scanning tool was used to determine the CMKLR1 and GPR1 mRNA expression in neuroblastoma. All expression data were scanned to find the most optimal cut-off between high and low gene expression and the log-rank test that gave the lowest p-value was calculated to search for significant differences between tumor samples expressing high and low CMKLR1 and GPR1 mRNA levels, respectively. The expression of *CMKLR1* **(C)** and *GPR1* **(F)** was compared between neural crest (Etchevers n=5), benign neurofibroma (Miller n=86) and 4 neuroblastoma cohorts (cohort 1: Versteeg n=88, cohort 2: Delattre n=64, cohort 3: Hiyama n=51, cohort 4: Lastowska n=30).

To investigate *CMKLR1* and *GPR1* gene expression in neuroblastoma we used the publically available R2: Genomics analysis and visualization platform http://r2.amc.nl. Examining two neuroblastoma gene expression cohorts, we found a correlation between high expression of *CMKLR1* (Figure 1A and 1B) and *GPR1* (Figure 1D and 1E) and a decrease in overall survival probability. Furthermore, *CMKLR1* expression was higher in neuroblastoma cohorts compared to benign neurofibroma and neural crest cells (Figure 1C). However, no difference was found comparing *GPR1* expression in the different cohorts (Figure 1F).

Additionally, we observed that expression of chemerin receptor *CCRL2* was elevated in the neuroblastoma cohorts compared to neurofibroma and neural crest, and that high expression of *CCRL2* correlated with a poor survival prognosis (Supplementary Figure 1D-1F). While chemerin (*RARRES2*) expression was higher in the neuroblastoma cohorts compared to the neural crest, no difference was seen in comparison with benign neurofibroma. Furthermore, no clear correlation between high expression of *RARRES2* and a decrease in overall survival probability was apparent due to conflicting results in the selected data sets (Supplementary Figure 1A-1C).

### Neuroblastoma cell lines express chemerin, CMKLR1 and GPR1

**Figure 2:**
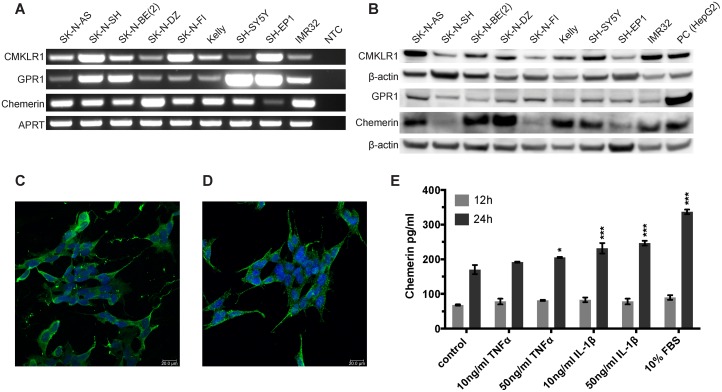
CMKLR1, GPR1 and chemerin are expressed in neuroblastoma cell lines and TNFα, IL-1β, and serum stimulate chemerin secretion **(A)** RT-PCR analysis demonstrating the expression of chemerin, CMKLR1 and GPR1 mRNA in all neuroblastoma cell lines investigated. NTC, no template control. The expression of chemerin, CMKLR1, and GPR1 protein was confirmed by western blot **(B)**. HepG2 cells were used as a positive control. The images are representative of three independent experiments. Immunofluorescence labeling shows the presence of CMKLR1 **(C)** and GPR1 **(D)** in SH-SY5Y cells (green). The nuclei (blue) were stained with Hoechst 33342, scale bar 20μm. **(E)** Chemerin concentrations were measured in cell supernatants of SK-N-AS cells after treatment with 10 or 50ng/ml TNFα, IL-1β or 10% FBS for 12 or 24h, respectively. The supernatants of 10 independent samples were pooled and concentrated 10x prior to ELISA measurement. The standards and samples were measured in duplicates and the data is presented as mean and range. Statistical analysis was performed using a two-way ANOVA P<0.001 for both stimulation and incubation time followed by Dunnett's post-test control vs. treatment ^*^ P<0.05, ^***^ P<0.001.

We examined different neuroblastoma cell lines for the expression of CMKLR1, GPR1 and chemerin. Using RT-PCR (Figure 2A) and western blot (Figure 2B) we demonstrated expression of CMKLR1, GPR1 and chemerin mRNA and protein at varying levels in all neuroblastoma cell lines tested. No correlation was apparent between CMKLR1, GPR1 or chemerin expression levels and specific cell line characteristics such as *MYCN* amplification, 1p deletion, 11q deletion or multi-drug resistance phenotype.

HepG2 cells were included in the western blots as a positive control. They are known to express and secrete chemerin and several antibody suppliers recommended them as a control cell line for CMKLR1.

Furthermore, we examined the expression levels of *RARRES2* (chemerin), *CMKLR1* and *GPR1* in a panel of neuroblastoma cell lines using the publically available R2: Genomics analysis and visualization platform http://r2.amc.nl. We observed that all three genes are expressed at varying levels in the neuroblastoma cell lines included in this panel (Supplementary Figure 2A-2C). In addition we compared their expression to known neuroblastoma promoting cytokines, chemokines, growth factors and their receptors and found *GPR1* and *CMKLR1* expression in the range of *FPR1*, *IL6R* and *PDGFRA.* While *RARRES2* (chemerin) expression is lower than *VEGFA*, it is comparable to *CCL2* and *CCL5* expression (Supplementary Figure 2D and 2E).

Immunofluorescence staining demonstrated the cellular distribution of CMKLR1 (Figure 2C) and GPR1 (Figure 2D) in the neuroblastoma cell line SH-SY5Y. Both receptors were localized at the cell membrane and in the cytoplasm. Comparable staining pattern for CMKLR1 was observed in other neuroblastoma cell lines using additional primary antibodies for confirmation (Supplementary Figure 3A and 3B). No apparent staining was observed in cells incubated with an isotype control antibody instead of the primary antibody (Supplementary Figure 3C).

### TNFα, IL-1β and serum increase chemerin secretion in neuroblastoma cells

To investigate the effect of the pro-inflammatory cytokines TNFα and IL-1β as well as serum components on chemerin expression and secretion, chemerin concentrations were measured by ELISA. Exposure to IL-1β, TNFα as well as 10% serum for 24h increased the level of chemerin in the supernatant of SK-N-AS cells (Figure 2E).

### CMKLR1, GPR1 and chemerin are expressed in neuroblastoma primary tumors

**Figure 3:**
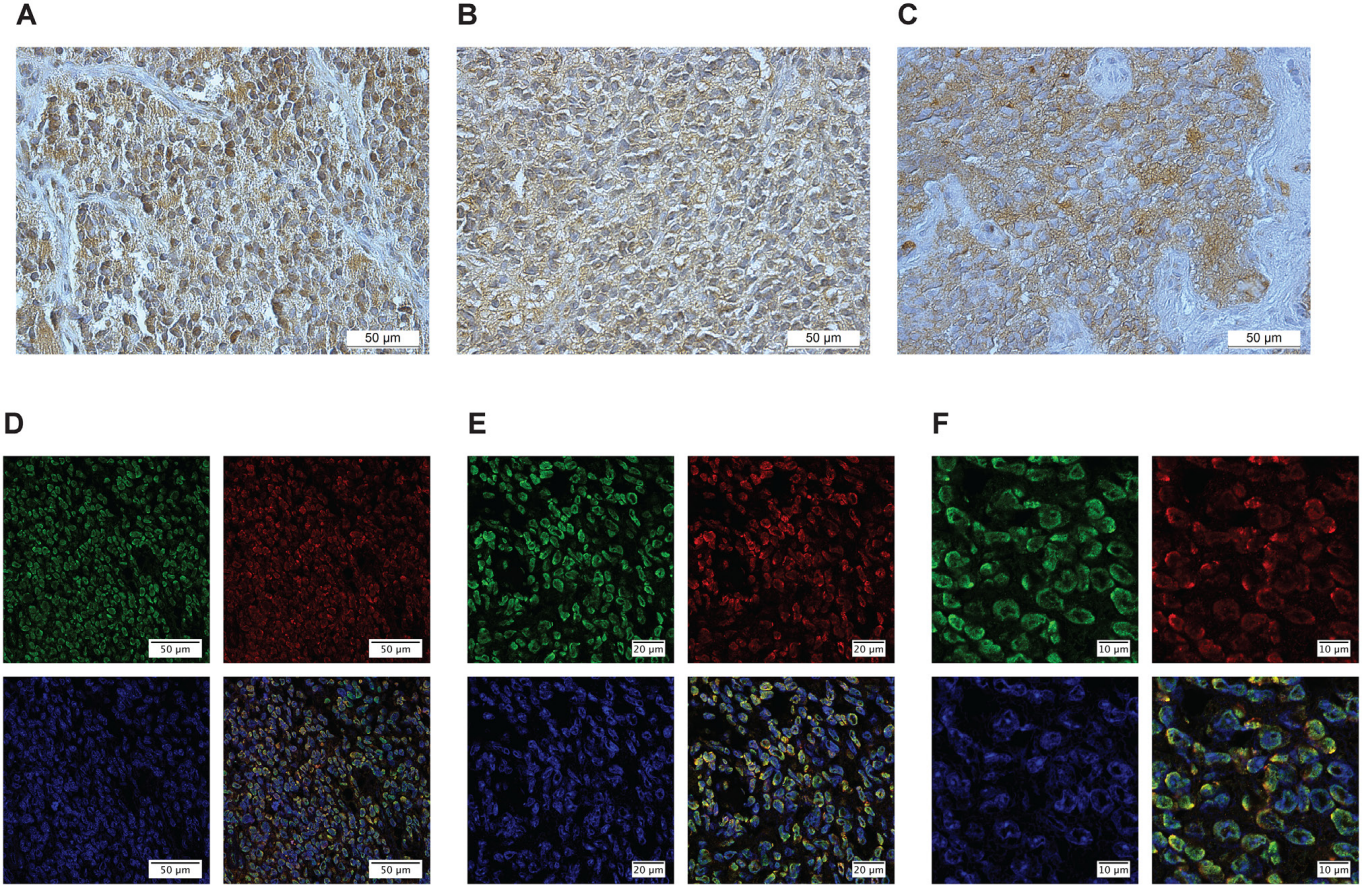
CMKLR1, GPR1 and chemerin are expressed in neuroblastoma primary tumors Immunoperoxidase staining demonstrates specific expression of **(A)** CMKLR1, **(B)** GPR1 and **(C)** chemerin in neuroblastoma primary tumor tissue. Immunofluorescence labeling **(D-F)** displays CMKLR1 (green) and chemerin (red) localization in neuroblastoma tissue. The nuclei were stained with DAPI (blue). (E and F) are higher magnifications of (D) to illustrate the colocalization of CMKLR1 and chemerin. The displayed images are representative stainings from a panel of neuroblastoma tumors.

To confirm the presence of CMKLR1, GPR1 and chemerin in neuroblastoma primary tumors, IHC and IF-P were performed. A total of 27 neuroblastoma tissue samples from all clinical stages and biological subsets [36] were stained with antibodies detecting chemerin, CMKLR1 and GPR1. All tumor samples investigated demonstrated a significant expression of chemerin and its receptors. Figure 3 presents a representative labeling with CMKLR1 (Figure 3A), GPR1 (Figure 3B) and chemerin (Figure 3C) specific antibodies showing a clear staining of both the receptors and chemerin in neuroblastoma primary tumors. Fluorescence labeling of CMKLR1 (green) and chemerin (red) displayed the membranous and cytoplasmic localization of CMKLR1 whereas chemerin was detected both in intra- and extracellular compartments (Figure 3D-3F) indicating chemerin secretion in neuroblastoma primary tumor tissue. For both, IHC and IF-P, no apparent staining was observed in sections incubated with isotype control antibodies instead of the primary antibodies (Supplementary Figure 3D-3F).

### Chemerin induces calcium mobilization and promotes MAPK and Akt signaling in neuroblastoma cell lines

Chemerin has been previously shown to stimulate intracellular calcium mobilization in immature DCs and macrophages as well as MAPK and Akt signaling in human chondrocytes and endothelial cells through CMKLR1 [14, 24, 37]. GPR1-mediated calcium mobilization and ERK1/2 phosphorylation following chemerin binding has been demonstrated to be much weaker [38, 39]. Recently, both CMKLR1 and GPR1 were found to signal through the RhoA/Rock pathway in HEK293A and gastric carcinoma cells [40].

**Figure 4:**
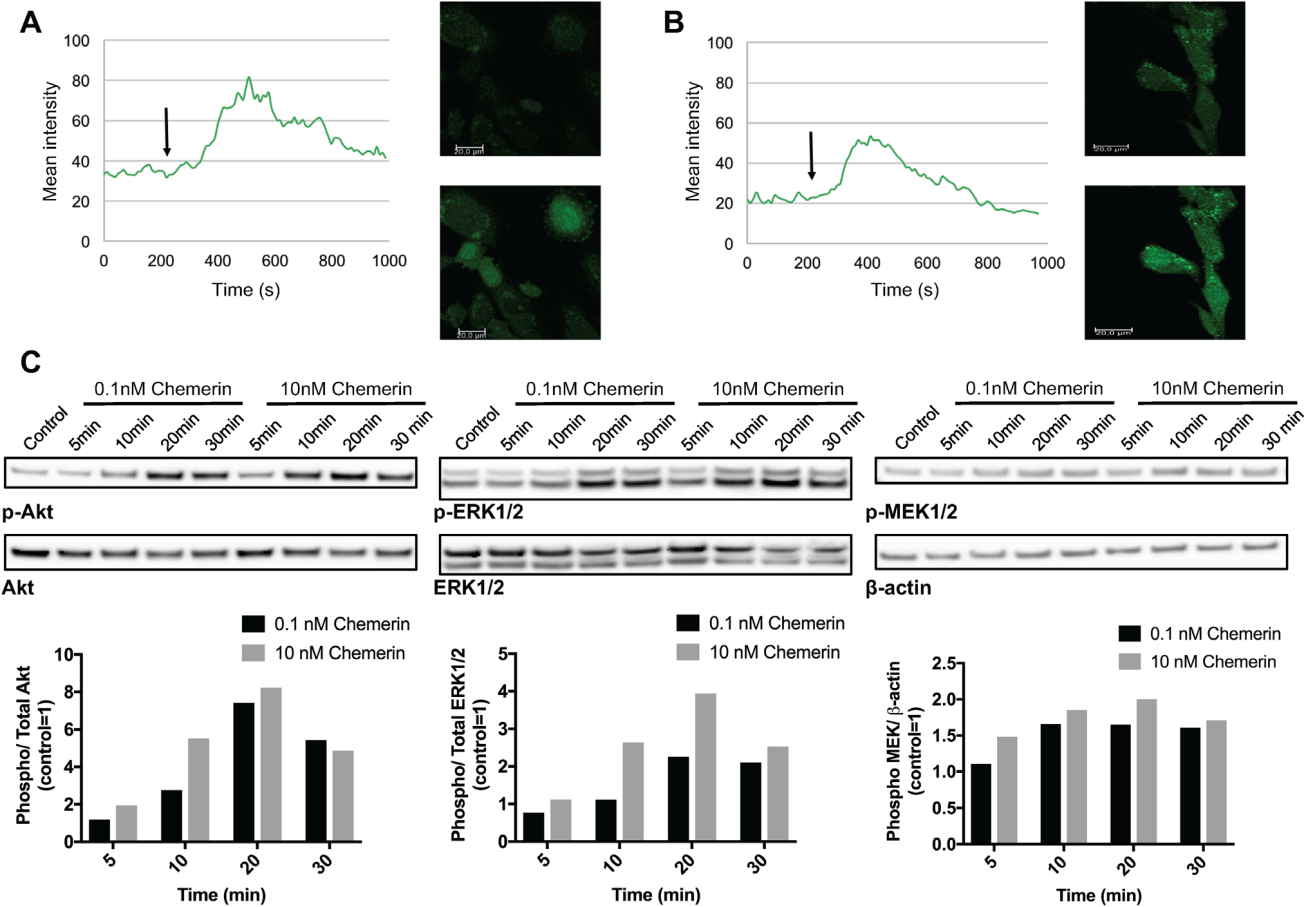
Chemerin induces intracellular calcium mobilization and stimulates MAPK and Akt signaling in neuroblastoma cells Intracellular calcium mobilization was measured in SK-N-SH cells with confocal laser scanning microscopy following the stimulation with 10nM chemerin without **(A)** and with **(B)** the prior addition of the calcium chelator EDTA. The arrow indicates the time point when chemerin was added. **(C)** Chemerin stimulates the phosphorylation of Akt, ERK1/2 and MEK1/2 in SK-N-AS cells in a dose-dependent manner. The cells were serum-starved for 24h prior to stimulation and samples were taken 5, 10, 20 and 30min after stimulation. Densitometric analysis of the protein bands was performed and the ratios between p-ERK1/2 and total ERK1/2, p-Akt and total Akt as well as p-MEK1/2 and β-actin were calculated. The values are displayed relative to the control=1. The experiments were performed three times with similar results.

To determine the effect of chemerin in neuroblastoma, we studied calcium mobilization, MAPK, and Akt signaling in neuroblastoma cell lines. Chemerin stimulation caused a rapid, but transient increase in intracellular calcium in SK-N-SH cells (Figure 4A and 4B) in comparison to vehicle treatment. Furthermore, prior incubation with the calcium chelator EDTA showed no inhibitory effect (Figure 4B) indicating calcium release from intracellular compartments.

The addition of chemerin to SK-N-AS cells induced a rapid and dose-dependent phosphorylation of MEK1/2, ERK1/2 and Akt (Figure 4C) indicating the activation of MAPK and Akt signaling. Similar phosphorylation patterns were observed in SK-N-BE(2) cells (data not shown).

### Chemerin increases MMP-2 synthesis in neuroblastoma cells

**Figure 5:**
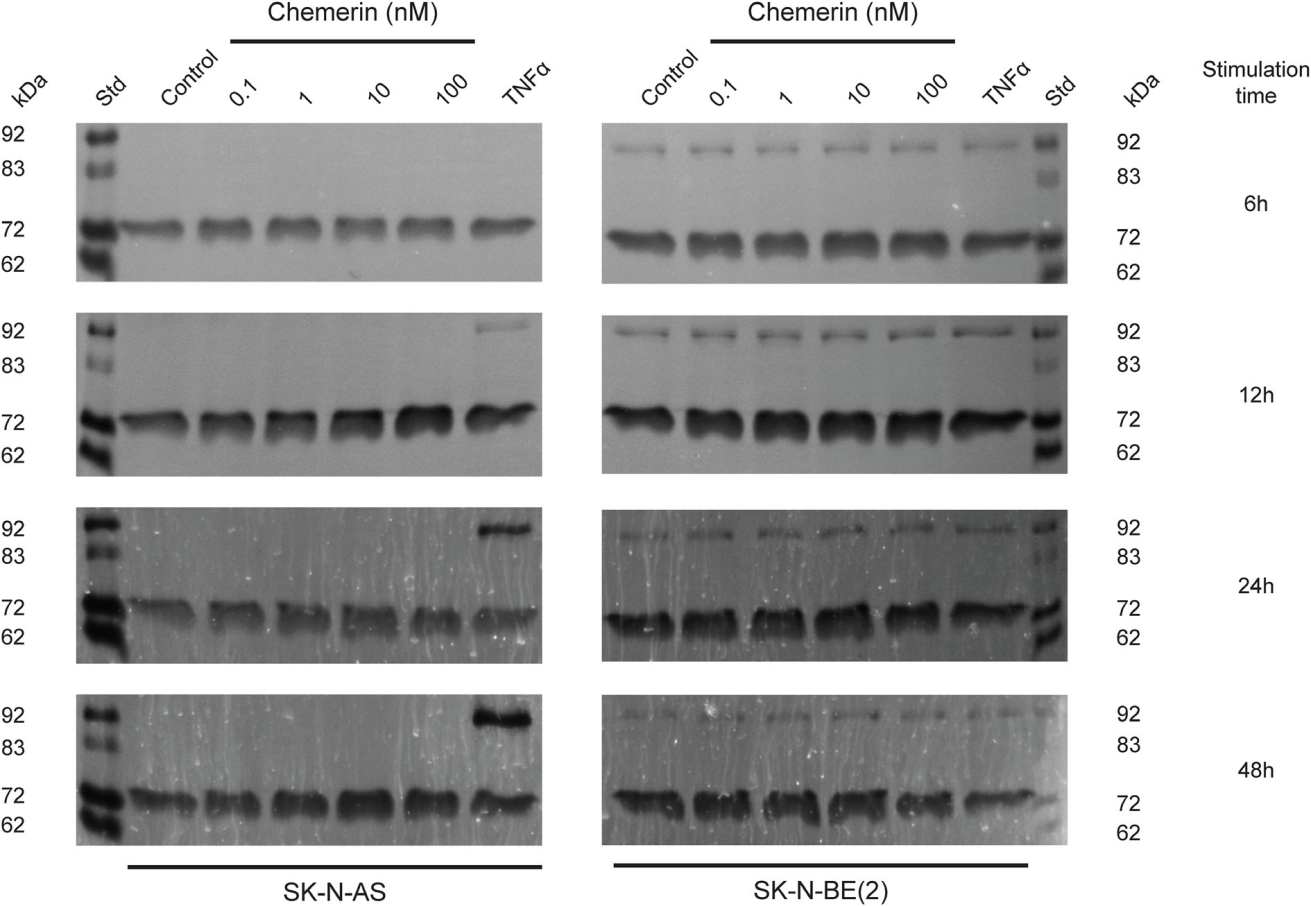
Chemerin stimulates MMP-2 synthesis in neuroblastoma cells Typical real-time gelatin zymography of supernatants from SK-N-AS and SK-N-BE(2) cells untreated (control) or treated with increasing concentrations of chemerin (0.1-100 nM) or TNFα (10 ng/ml) for 6h, 12h, 24h and 48h. Prior to stimulation, the cells were serum-starved for 24h. For each zymogram the supernatants of three independent samples were pooled and analyzed. The shown zymograms were taken after optimal incubation time (for SK-N-AS 15h, 10h, 13h and 13h and for SK-N-BE(2) 15h, 10h, 5h and 4h) in assay buffer after the removal of SDS from the gels. The standard (st) comprised a mixture of proMMP-9 monomer (92 kDa), active MMP-9 (83 kDa), proMMP-2 (72 kDa) and active MMP-2 (62 kDa).

Chemerin is known to stimulate MMP-2 and MMP-9 expression and activity [37, 41]. Using real-time zymography, we could follow the degradation of gelatin by MMP-2 and MMP-9 in real-time. We observed a dose-dependent increase in MMP-2 synthesis in both SK-N-AS and to a lesser extent in SK-N-BE(2) cells after 6, 12, 24 and 48h stimulation with active chemerin (Figure 5). No effect on MMP-9 synthesis was observed under these conditions.

### CMKLR1 inhibition reduces the cell viability and clonogenicity of neuroblastoma cells

**Figure 6:**
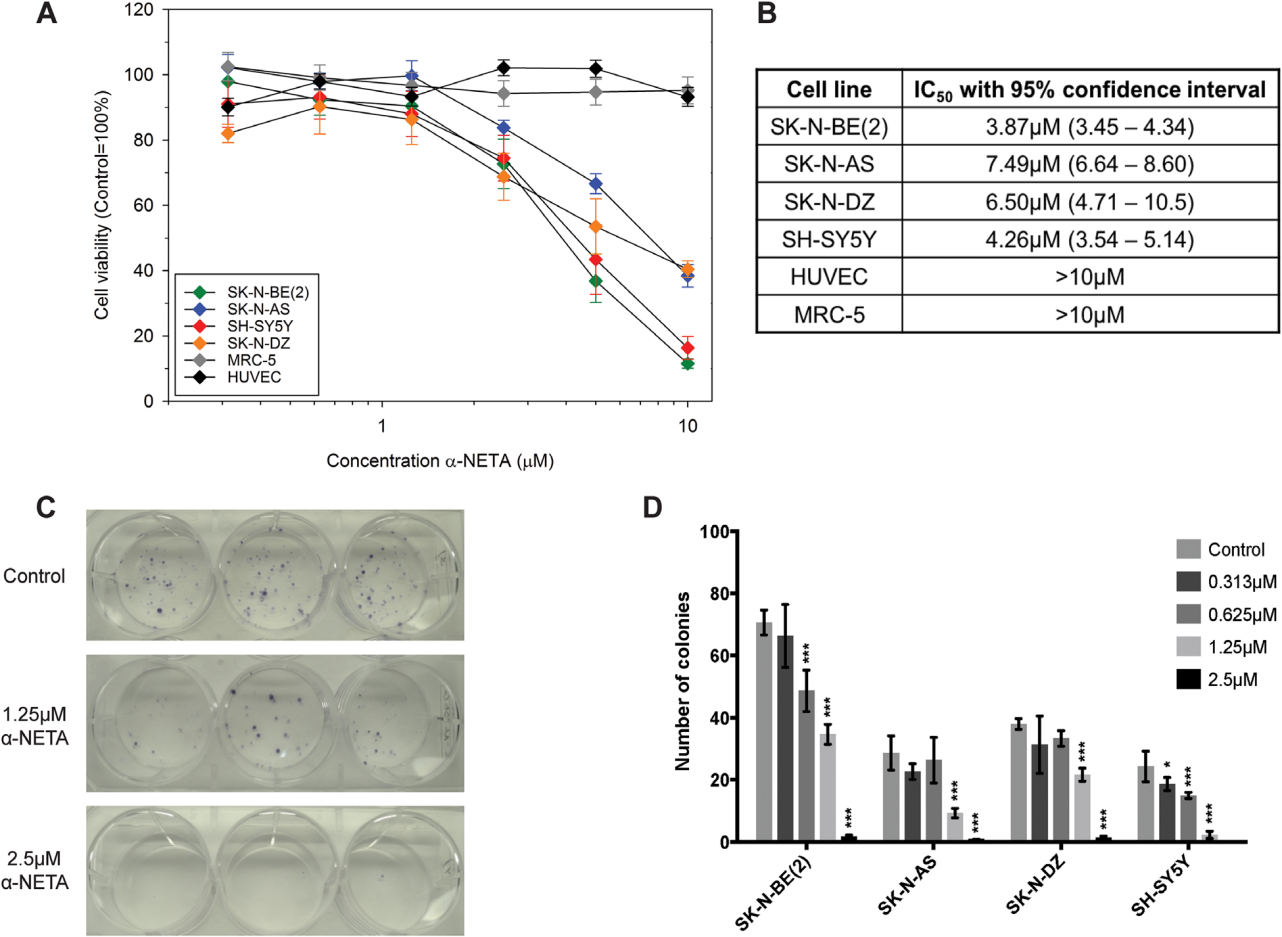
The CMKLR1 antagonist α-NETA reduces the cell viability and clonogenicity of neuroblastoma cell lines Cell viability was determined after 72h of incubation with α-NETA (0.313-10μM). A dose-dependent decrease in cell viability was observed in the neuroblastoma cell lines but not in MRC-5 and HUVEC cells **(A)**. Data is presented as mean ± SEM from three experiments. The IC_50_ values are given with 95% confidence intervals in **(B)**. The mean of log IC_50_s in neuroblastoma cell lines was significantly lower than the hypothetical log IC_50_ of the investigated normal cell lines (one sample t-test, p=0.029; means: 0.726 vs 1). The CMKLR1 antagonist α-NETA reduces the clonogenicity of SK-N-BE(2) cells (**C**; 1.25 and 2.5μM, n=3) and other neuroblastoma cell lines (**D**; 0.313-2.5μM, n=3) in a dose-dependent manner after 72h treatment. Data is presented as mean ± SD from a representative experiment. The experiment was repeated twice more with similar results. Statistical testing was performed using a two-way ANOVA P<0.001 for both stimulation and between cell lines followed by Dunnett's post-test control vs. treatment ^*^ P<0.05, ^***^ P<0.001.

The effect of CMKLR1 inhibition on neuroblastoma cell lines was studied using the recently described CMKLR1 inhibitor α-NETA [42]. Increasing concentrations of α-NETA reduced the cell viability of four neuroblastoma cells lines after 72h of treatment with IC_50_ values ranging between 3.87-7.49μM (Figure 6A and 6B). No effect (IC_50_ values >10μM) was observed on human fibroblasts (MRC-5) and endothelial cells (HUVEC). A dose-dependent inhibition of clonogenicity was observed in SK-N-BE(2) cells (Figure 6C) as well as SK-N-AS, SK-N-DZ, and SH-SY5Y cells (Figure 6D). The colony forming ability was completely inhibited using 5μM α-NETA.

### Early and prolonged CMKLR1 inhibition impairs neuroblastoma growth *in vivo*

**Figure 7:**
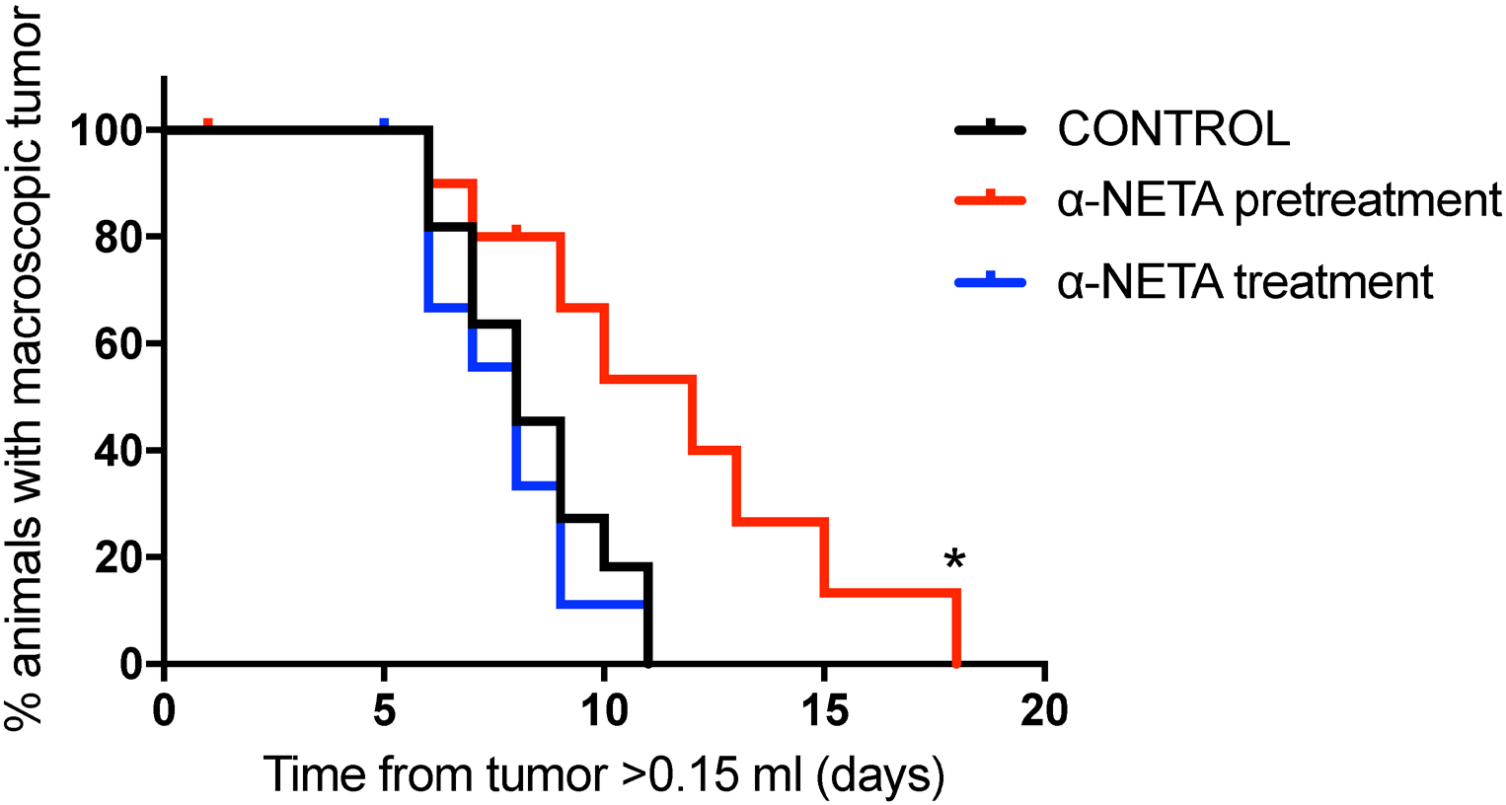
Early and prolonged CMKLR1 inhibition with α-NETA impairs tumor growth *in vivo* Kaplan-Meier survival plots of nude mice (n=11 in control group and in pre-treatment group, n=10 in treatment group) injected daily s.c. with 20mg/kg α-NETA after the tumor reached 0.15ml (treatment group), 10mg/kg α-NETA from the day after tumor cell injection and 20mg/kg when the tumor reached 0.15ml (pre-treatment group) or 10% Captisol^®^ (control group). Survival, defined as time needed for the animals to grow a macroscopic tumor (volume >1.5ml), was significantly prolonged in the pre-treatment group (log rank test P=0.015, control vs. pre-treatment P= 0.019 and control vs. treatment P=0.51).

The therapeutic effect of CMKLR1 inhibition was examined in a SK-N-AS xenograft model. A significant prolongation (p=0.015, Log rank test) of survival (defined as time needed for the animals to grow a macroscopic tumor with a volume >1.5ml) was observed in the pre-treatment group, where the animals were treated s.c. with α-NETA continuously from day 1 after tumor cell injection, compared to the control group (Figure 7). In addition, when comparing tumor growth rates for individual tumors, the tumors from the pre-treated mice grew significantly slower than the tumors in the control group (p=0.0061, one way ANOVA with Bonferroni post-test, control vs. pre-treatment p=0.049, Supplementary Figure 4). However, no effect was seen in the treatment group, where α-NETA s.c. injections were initiated after the tumor reached a volume of ≥ 0.15ml, compared to the control group. No signs of toxicity were observed at the used concentrations of α-NETA. In the pre-treatment group, a hardening of the skin was seen at the later stages of the experiment probably due to the daily s.c. injection over a prolonged period. All mice gained weight over the course of the experiment.

## DISCUSSION

Neuroblastoma is a malignancy with only a few identified key genetic events. Besides amplification of the *MYCN* oncogene (in approximately 20% of neuroblastomas), *ALK* mutations and amplifications occur in 9% and 2-3%, respectively. Other affected genes include *ODC, NTRK2/TrkB, FOXR1, PTPN11, ATRX, CADM1*, and *ARID1A/B* [2, 43]. For the discovery of potential new therapeutic targets, understanding of the neuroblastoma TME is of great importance. Chemokines and chemoattractant factors are essential regulators of cell trafficking during immune response and inflammation. Furthermore, they are involved in all stages of cancer development: tumor establishment, neovascularization, and metastasis [44, 45].

Several chemokine receptors and their ligands have been identified as contributors to neuroblastoma angiogenesis, metastasis, and communication between tumor cells and cells of the TME [6, 46]. CMKLR1 is a chemoattractant receptor present on immune cells such as immature DCs, macrophages and NK cells [47]. Recently, CMKLR1 was found to be expressed in a subset of myeloid-derived suppressor cells (MDSCs) in hepatocellular carcinoma [48].

Chemerin, a ligand for CMKLR1 possesses a wide variety of characteristics attributed to tumor growth such as chemotaxis and cell adhesion, as well as cell survival and proliferation [21, 41, 47]. In ECs, CMKLR1 was found to be upregulated by the pro-inflammatory cytokines TNFα, IL-1β and IL-6. Furthermore, chemerin stimulation induced MMP production and angiogenesis [37]. Recent work by Chua et al. demonstrates that hypoxia promotes chemerin expression in human coronary artery endothelial cells as well as migration and tube formation, supporting previous findings on the role of chemerin in angiogenesis [49]. However, the function of the chemerin/CMKLR1 axis in malignancies is probably tumor specific as both tumor promoting and tumor suppressing roles have been reported [15–18, 20, 30, 31].

In the present study, we investigated the role of chemerin and its receptors CMKLR1 and GPR1 in neuroblastoma tumorigenesis. Using publically available gene expression datasets (http://r2.amc.nl) we observed that high *CMKLR1* and *GPR1* expression correlates with a reduced overall survival probability in the two datasets we examined. We did not find any relationship between genetic characteristics of neuroblastoma, such as MYCN expression, and CMKLR1, GPR1 or chemerin expression. Our findings indicate that CMKLR1 and/or GPR1 could potentially be used as independent prognostic factors.

Tumor-associated macrophages (TAMs) have been shown to promote neuroblastoma tumorigenesis [6], and CMKLR1 expression in macrophages can be stimulated by mammary and lung carcinoma cells [50]. Asgharzadeh et al. [7] described the prognostic value of a TAM gene expression signature (*CD33, CD16, IL6R, IL10, FCGR3*) in metastatic neuroblastoma. Examining publically available neuroblastoma gene expression datasets (R2: Genomics Analysis and Visualization Platform http://r2.amc.nl) we observed a significant correlation between expression of CMKLR1 and the TAM markers (Supplementary Figure 5). IHC labeling of the macrophage marker CD68 as well as double IF-P staining of CD68 and CMKLR1 in neuroblastoma tissue demonstrated that while the majority of CMKLR1 is expressed by the tumors cells, CD68^+^ cells in the TME also express CMKLR1 (Supplementary Figure 6).

Additionally, CMKLR1 is expressed at high levels in monocytic MDSCs in hepatocellular carcinoma [48]. Therefore, the expression of CMKLR1 on tumor promoting immune cells could also play a significant role in the tumorigenesis process.

A recent study demonstrated that chemerin secreted by esophageal squamous cancer-associated myofibroblasts stimulates the migration of cancer cells, indicating a role in invasion. Blockage of the chemerin/CMKLR1 axis inhibited invasion [41]. In addition, Kaur et al. determined a mitogenic effect of chemerin on human macro- and microvascular endothelial cells through activation of CMKLR1 [37]. Therefore, a potential role of chemerin/CMKLR1 in neuroblastoma angiogenesis could be hypothesized. Collectively, these findings indicate that targeting CMKLR1 expressed on stromal cells in addition to the tumor cells could be of therapeutic interest.

In the present study, active chemerin induced both calcium mobilization and the activation of MAPK and Akt signaling. PI3K/Akt- and MAPK-mediated signaling are known to contribute to neuroblastoma tumorigenesis [51–54]. Furthermore, we demonstrated that the pro-inflammatory cytokines TNFα and IL-1β as well as serum components stimulate chemerin secretion by neuroblastoma cells. Both cytokines have been previously found to regulate CMKLR1 and chemerin expression in human endothelial cells, keratinocytes (IL-1β), and other cell types [37, 55]. Additionally, we observed that chemerin stimulated MMP-2 synthesis in a dose-dependent manner. MMP-2 is a member of the matrix metalloproteinase family with important functions in tumorigenesis. Through processing of extracellular matrix and non-matrix proteins, MMP-2, and other members of the MMP family, contribute to cell invasion, metastasis and neovascularization [56]. Increased MMP-2 expression in neuroblastoma has been associated with increased angiogenesis, advanced stage, and poor clinical outcome [57, 58]. Our results indicate that chemerin may contribute to an increased MMP-2 synthesis in neuroblastoma.

In our work, we observed that α-NETA, a small molecule inhibitor for CMKLR1, reduced the cell viability and clonogenicity of neuroblastoma cell lines. Initially identified as a choline acetyltransferase inhibitor, α-NETA was recently found to be a more potent inhibitor of CMKLR1 [42]. In an *in vivo* xenograft study, we observed that continuous, long-term treatment with α-NETA resulted in impaired tumor growth. However, no effect was observed when α-NETA injections were initiated after the tumor had reached a volume of 0.15ml. These results indicate that CMKLR1 function might be of greater importance during the early stages of tumor growth as well as tumor recurrence and relapse in neuroblastoma patients. However, SK-N-AS xenograft tumors grow very fast once the tumor has been established therefore providing only a narrow treatment window. In order to achieve a significant effect, a longer treatment window as given in the pre-treatment group might be necessary. The results from the *in vitro* studies indicate a role of CMKLR1 during clone formation. Inhibiting CMKLR1 at a stage where the tumor has reached a certain size might therefore have a smaller impact. Since α-NETA has only recently been described as a CMKLR1 inhibitor, potential off-target effects are not fully studied. Concerning bioavailability and stability of α-NETA *in vivo* only limited data is available, hampering dose estimation. Hence, the concentration used in this study might have been too low to sufficiently abrogate CMKLR1 function in established tumors. Further studies are therefore necessary to determine the appropriate concentrations for α-NETA *in vivo*. Additionally, the results should be confirmed using *CMKLR1* knockout neuroblastoma cell lines. Although we made several attempts to knock down/out CMKLR1 in neuroblastoma cell lines using both shRNA and the CRISPR/Cas9 system, we have established to date only one SK-N-AS cell line with a marked CMKLR1 downregulation (Supplementary Figure 7). Since this cell line grows very slowly and is unable to form distinct colonies in clonogenicity assays we have been unable to utilize it in functional *in vitro* or *in vivo* studies. While these findings, taken together with the results from the inhibitor studies, indicate that CMKLR1 may contribute to colony formation and tumorigenesis we have been unable to confirm these findings with additional knock down clones.

GPR1 is an additional active chemerin receptor expressed in the central nervous system, skeletal muscle, and adipose tissue [47]. In this work, we also demonstrate the expression of GPR1 mRNA and protein in neuroblastoma cell lines and primary tumor tissue. While most of the known chemerin functions have been connected to CMKLR1-mediated signaling, we cannot exclude that chemerin mediated signaling in neuroblastoma cell lines is not at least partly mediated by GPR1. However, GPR1 mediated calcium mobilization and ERK1/2 phosphorylation has been demonstrated to be much weaker compared to CMKLR1 [38, 39]. Recently, chemerin was found to activate RhoA/Rock signaling through CMKLR1 and GPR1 [40]. The Rho/Rock pathway is involved in actin rearrangement, hence suggesting a potential role of the chemerin/CMKLR1/GPR1 axis in migration and metastasis.

CCRL2, the third known chemerin receptor is present on myeloid cells, mast cells and CD34+ bone marrow precursors [47]. While not actively signaling, it was found to increase local chemerin levels suggesting that CCRL2 presents chemerin to CMKLR1 or GPR1 on neighboring cells [33]. Akram et al. recently identified a role of CCRL2 in colorectal cancer liver metastases [35]. Although we were able to detect CCRL2 mRNA in neuroblastoma cell lines (data not shown), the role of CCRL2 in neuroblastoma was not addressed in the present study.

Our results demonstrate, for the first time, the presence of a fully active and functional chemerin/CMKLR1 axis in childhood neuroblastoma. Neuroblastoma cells produce chemerin that can promote survival in an autocrine manner. Inhibition of the chemerin/CMKLR1 axis impaired neuroblastoma cell growth *in vitro* and *in vivo*. Our findings provide new insight into the pathobiology of neuroblastoma. Pharmacological interventions targeting the chemerin/CMKLR1 signaling pathway may be an important adjuvant therapy for children with neuroblastoma, but further preclinical *in vivo* studies are warranted.

## MATERIALS AND METHODS

### Microarray gene expression analysis

Gene expression analysis was performed using the publicly available R2: Genomics Analysis and Visualization Platform (http://r2.amc.nl).

### Antibodies and reagents

The antibodies used in this study are listed in Table 1. Recombinant human IL-1β was purchased from Cell Guidance Systems Ltd. (Cambridge, UK). Recombinant human TNFα and chemerin were obtained from R&D Systems, Inc. (Minneapolis, USA) and α-NETA was from Santa Cruz Biotechnology, Inc. (Dallas, USA).

**Table 1:** Antibodies used in the study

Antibody	Application	Source
Anti-ChemR23	WB, IHC	#STJ92262, St John's Laboratory
Anti-Human ChemR23	IF-P, ICC	#MAB362, R&D Systems
Anti-GPCR GPR1	WB	#ab157209, abcam
Anti-GPCR GPR1	ICC	#ab121315, abcam
Anti-GPCR GPR1	IHC	#ab188977, abcam
Anti-TIG2 Antibody (K-15)	WB, IF-P	#sc-47482, Santa Cruz Biotechnology
Anti-Human Chemerin	IHC	#MAB2324, R&D Systems
Anti-beta Actin	WB	#ab8227, abcam
Anti-p44/42 MAPK (Erk1/2)	WB	#4695, Cell Signaling Technology
Anti-Phospho-p44/42 MAPK (Erk1/2) (Thr202/Tyr204)	WB	#4370, Cell Signaling Technology
Anti-Akt	WB	#9272, Cell Signaling Technology
Anti-Phospho-Akt (Ser473) (D9E)	WB	#4060, Cell Signaling Technology
Anti-Phospho-MEK1/2 (Ser217/221)	WB	#9121, Cell Signaling Technology
Goat Anti-Rabbit IgG H&L (HRP)	WB	#ab6721, abcam
Swine Anti-Goat Ig's, HRP	WB	#ACI0404, Thermo Fisher Scientific
Goat anti-Rabbit IgG (H+L), Alexa Fluor 488	ICC	# A-11008, Thermo Fisher Scientific
Donkey anti-Goat IgG (H+L), Alexa Fluor 594	IF-P	#A-11058, Thermo Fisher Scientific
Rabbit anti-Mouse IgG (H+L), Alexa Fluor 488	IF-P	# A-11059, Thermo Fisher Scientific

### Cell lines and human tissue samples

The human neuroblastoma cell lines SK-N-AS, SK-N-SH, SK-N-DZ, SK-N-FI, SH-EP1, Kelly, SH-SY5Y, and IMR-32 as well as the hepatocellular carcinoma cell line HepG2 were purchased from the ATCC (American Type Culture Collection), and SK-N-BE(2) cells were bought from DSMZ (Deutsche Sammlung von Mikroorganismen und Zellkulturen). The cells were cultivated in RPMI-1640 medium containing L-glutamine and sodium bicarbonate (Sigma-Aldrich Norway AS, Oslo, Norway) supplemented with 10% heat-inactivated FBS (Thermo Fisher Scientific Inc.) at 37°C in humidified air with 5% CO_2_. The human fibroblast cell line MRC-5 and human umbilical vein endothelial cells (HUVEC) were purchased from the ATCC and cultivated in EGM-2 BulletKit with 2% FBS (Lonza, Basel, Switzerland) and MEM supplemented with 2mM L-glutamine, 1% non-essential amino acids and 10% FBS, respectively. Mycoplasma tests were regularly performed using the MycoAlert™ PLUS Mycoplasma Detection Kit (Lonza, Basel, Switzerland).

Neuroblastoma tumor tissue was obtained from the Karolinska University Hospital according to the ethical approval from the Stockholm Regional Ethical Review Board and the Karolinska University Hospital Research Ethics Committee (approval ID 2009/1369-31/1 and 03-736). Informed consent (written or verbal) was provided by the parents or guardians for the use of tumor samples in research. Samples were collected during surgery, snap-frozen in liquid nitrogen and stored at -80°C until further use. Twenty-seven neuroblastoma samples derived from children of different ages and all clinical stages, including different biological subsets [36] were analyzed.

### RNA isolation and reverse transcriptase PCR

Total RNA was isolated using the RNeasy^®^ Mini Kit (Qiagen Norge, Oslo, Norway) according to the provided manual. The RNA quantity and quality was determined using the NanoDrop 1000 (Thermo Fisher Scientific Inc.). One μg RNA was used for cDNA synthesis with the iScript™ cDNA Synthesis Kit (Bio-Rad Laboratories AB, Oslo, Norway). PCR was performed in a 25μl reaction mix containing 2μl cDNA, 12.5μl AccuStart™ II GelTrack PCR SuperMix (Quanta Biosciences, Gaithersburg, USA), 400 nM of each primer and 10.1μl of ultra-pure H_2_O (Biochrom GmbH, Berlin, Germany). The PCR run was performed in a T100™ Thermal Cycler (Bio-Rad Laboratories AB, Oslo, Norway) as follows: 2min at 94°C and 35 cycles of 94°C for 20s, 62°C for 30s and 72°C for 90s. The sequences for the PCR primers were the following: APRT (housekeeping) 5’-CCCGAGGCTTCCTCTTTGGC-3’ (sense) and 5’-CTCCCTGCCCTTAAGCGAGG-3’ (antisense) [59], CMKLR1 5'-GCCAACCTGCATGGGAAAATA-3’ (sense) and 5’-GTGAGGTAGCAAGCTGTGATG-3’ (antisense), GPR1 5’-CAATCTAGCCATTGCGG ATTTCA-3’ (sense) and 5’-CCGATGAGATA AGACAGGATGGA-3’ (antisense), chemerin 5’-AGAAACCCGAGTG CAAAGTCA-3’ (sense) and 5’-AGAACTTGGGTCTCTATGGGG-3’ (antisense) (Primer bank ID 215272316c3, 148228828c3 and 218931208c1 http://pga.mgh.harvard.edu/primerbank/index.html).

PCR products were analyzed by gel electrophoresis. The 1.8% SeaKem^®^ LE Agarose gel (Lonza) was stained with GelRed™ (Biotium, Inc., Hayward, USA) and visualized under UV light in the BioDoc-It™ Imaging System (UVP, LLC, Upland, USA). The PCR results for CMKLR1, GPR1 and chemerin were confirmed with a second, independent primer set (data not shown).

### Western blot

Cultured cells were washed briefly with phosphate-buffered saline (PBS, Biochrom GmbH) and harvested in RIPA Lysis and Extraction Buffer containing Halt™ Protease and Phosphatase Inhibitor Cocktail (Thermo Fisher Scientific Inc.). Following sonication, the protein concentration was determined using a Protein Quantification Assay (MACHEREY-NAGEL GmbH & Co. KG, Düren, Germany). The protein lysates were supplemented with NuPAGE^®^ LDS Sample Buffer (4X) (Thermo Fisher Scientific Inc.) as well as 100mM DTT (Sigma-Aldrich Norway AS) and incubated for 10min at 70°C. Equal amounts of protein were separated on NuPAGE™ Novex™ 4-12% Bis-Tris Protein Gels (Thermo Fisher Scientific Inc.) and transferred onto a 0.45μm PVDF Membrane (Merck Life Science AS, Oslo, Norway) according to the XCell SureLock Mini-Cell technical guide (Thermo Fisher Scientific Inc.). The membranes were blocked in TBS-T (Tris-buffered saline (TBS) with 0.1% Tween-20; Sigma-Aldrich Norway AS) containing 5% (w/v) skimmed milk powder. Incubation with the primary antibody was performed overnight at 4°C according to antibody supplier recommendation in either blocking buffer or 5% BSA (AppliChem, Darmstadt, Germany) in TBS-T. Following three washes in TBS-T, the membranes were incubated in the appropriate secondary antibody solution for 1h at room temperature. After four washes, detection and visualization were performed using SuperSignal™ West Pico Chemiluminescent Substrate (Thermo Fisher Scientific Inc.) and the ImageQuant LAS 4000 imager (GE Healthcare, Oslo, Norway). MagicMark™ XP Western Protein Standard (Thermo Fisher Scientific Inc.) was used to estimate the molecular mass of the detected proteins. Densitometry was performed using Fiji software [60].

### ICC

For immunocytochemistry, cells were grown on 8-well μ-Slide (ibidi GmbH, Munich, Germany) for 24h. Cells were then rinsed briefly with PBS and fixed with 4% formaldehyde for 20min. After three washes with PBS, unspecific binding sites were blocked with 1% BSA in PBS containing 0.3% Triton-X-100 (Sigma-Aldrich Norway AS) for 45min. The cells were incubated with primary antibodies diluted in blocking solution at 4°C overnight. After three washes with PBS, the cells were incubated with the secondary antibodies diluted in blocking solution for 1h at room temperature, protected from light. Following three washes with PBS, the nuclei were stained with Hoechst 33342 (ImmunoChemistry Technologies, LLC, Bloomington, USA) for 10min. The cells were washed 2x with PBS and covered with Mounting Medium for fluorescence microscopy (ibidi GmbH). The cells were subsequently examined with a Leica TCS SP5 or Zeiss LSM780 confocal microscope.

### IHC

Formalin-fixed and paraffin-embedded tissue sections were deparaffinized in xylene and graded alcohols, hydrated and washed in PBS. After antigen retrieval in a sodium citrate buffer (pH 6) in a microwave oven, the endogenous peroxidase was blocked by 0.3% H_2_O_2_ for 15min. Sections were incubated overnight at 4°C with the primary antibody. As a secondary antibody, the anti-rabbit-HRP SuperPicTure Polymer detection kit (87-9663, Zymed-Invitrogen, San Francisco, USA) or anti-mouse EnVision-HRP (Dako, Agilent Technologies, Inc., Santa Clara, USA) was used. A matched isotype control was used as a control for nonspecific background staining.

For immunofluorescence histology studies (IF-P), the sections were treated as described above and stained sequentially with the primary and secondary antibody sets. Alexa Fluor^®^ 488 and Alexa Fluor^®^ 594 conjugated secondary antibodies were used to visualize positive staining.

The fluorescence labeled tissue sections were examined using the Zeiss LSM780 confocal microscope and the immunoperoxidase stained sections using the Leica DMI6000B microscope.

### Calcium mobilization assay

SK-N-SH cells were seeded into an 8-well μ-Slide (ibidi GmbH) and incubated overnight in RPMI containing 10% FBS. The following day the cells were washed and preloaded with 20μM Cal-520 (AATBio, Sunnyvale, USA) in Hanks' Buffer with 20mM Hepes (HHBS) with 0.04% Pluronic^®^ F-127 (AATBio). After 90min of incubation at 37°C, the dye solution was replaced with HHBS and the cells were subsequently examined with a Leica TCS SP5 confocal microscope in the presence or absence 2mM EDTA. Before the addition of 10nM of chemerin, a baseline measurement was taken. Images were then obtained and analyzed using the Leica LAS AF software.

### Stimulation of cells with chemerin

Cells were seeded in 35mm cell culture dishes (Corning, Corning, USA) and incubated overnight in complete growth medium. The cells were serum starved for 24h prior to stimulation with recombinant human chemerin (0.1-10 nM) for 5, 10, 20 and 30min.

### Chemerin ELISA

SK-N-AS cells were seeded in 96-well culture plates. The following day, the medium was removed and the cells were serum starved (0.1% FBS) overnight. The cells were then stimulated with either 10% FBS or 10 ng/ml, 50 ng/ml TNFα, or IL-1β in serum reduced medium (0.1% FBS) for 12 and 24h. After incubation, supernatants from 10 parallels were pooled and spun for 5min at 200xg to pellet floating cells. The cell supernatant was concentrated 10x using Amicon Ultra-0.5 Centrifugal Filter Unit (Merck Life Science AS). The chemerin quantity was assayed by ELISA according to the manufacturer's instructions (Human Chemerin Quantikine ELISA, R&D Systems, Inc).

### Real-time zymography

SK-N-AS and SK-N-BE(2) cells were seeded in 96-well plates (Corning, Corning, USA) and left to attach overnight. The medium was replaced with Opti-MEM and the cells were serum-starved for 24h. The cells were thereafter exposed to chemerin (0.1-100 nM) for 6, 12, 24 and 48h, using Opti-MEM serum-free medium. TNFα (10ng/ml) was used as a positive control. After the incubation, the medium from three independent samples was pooled, centrifuged at 200xg for 5min at 4°C, and made 10 and 100mM with respect to CaCl_2_ and Hepes (pH 7.5). Undiluted samples were analyzed for the expression of gelatin degrading enzymes using real-time zymography. Zymography was performed as described previously [61] with the exception that 0.1% (w/v) 2-methoxy-2,4-diphenyl-3(2H)-furanone (MDPF)-fluorescent labeled gelatin was incorporated in the 7.5 % SDS-PAGE separating gel instead of 0.1% (w/v) unlabeled gelatin. Gelatin (Sigma-Aldrich Norway AS) was labeled with the fluorescent dye 2-methoxy-2,4-diphenyl-3(2H)-furanone (Sigma-Aldrich Norway AS) to give MDPF-gelatin as described previously [62]. The main difference between normal gelatin zymography and real-time gelatin zymography is that in real-time zymography the gel is not stained and hence it is possible to follow the degradation of the gelatin in real time without staining. In the present work, each gel was monitored continuously and a picture of the gel was taken approximately every second hour for fifteen hours or more. Gelatinase activity was evident as dark bands against the un-degraded fluorescent background.

### Cell viability assay

A colorimetric 3-(4,5-dimethylthiazol-2-yl)-2,5-diphenyltetrazolium bromide (MTT) viability assay [63] was employed to assess the effect of α-NETA on the viability of neuroblastoma cell lines as well as MRC-5 and HUVEC. The cells were seeded in 96-well plates in full growth media. After 24h the cells were washed once with Opti-MEM (Thermo Fisher Scientific Inc.) before being incubated with 313nM-10μM α-NETA (dissolved in dimethylsulfoxide, DMSO) in Opti-MEM for 72h. Control cells received DMSO corresponding to the highest concentration present in the α-NETA treated cells. The MTT solution (20μl of 5mg MTT, Sigma-Aldrich Norway AS, per ml phosphate buffered saline) was added to each well. After 2-3h additional incubation 150μl of solution were carefully removed from each well and 100μl isopropanol containing 0.04M HCl were added and mixed thoroughly. To further facilitate formazan crystal solubilizing, the plates were placed on an orbital shaker for 1h at room temperature. The absorbance was measured with a CLARIOstar plate reader (BMG LABTECH, Ortenberg, Germany) at 590 nm. The experiment was repeated three times with at least three parallels per treatment and the cell viability was calculated as the ratio of the mean OD of treated cells over vehicle treated control cells (100% living cells). The IC50s were calculated from log concentration curves using non-linear regression analysis in GraphPad Prism.

### Clonogenic assay

SK-N-AS, SK-N-BE(2), SK-N-DZ, and SH-SY5Y cells were seeded in 6 well plates and allowed to attach to the surface overnight. The cells were washed and the medium was replaced with Opti-MEM containing 313 nM-5μM α-NETA dissolved in DMSO. The control cells received DMSO corresponding to the highest concentration present in the α-NETA treated cells. After 72h the medium was replaced with regular growth medium containing 10% FBS. When the cell colonies reached ≥ 50 cells, the plates were briefly rinsed with PBS (Thermo Fisher Scientific Inc.), fixed in 4% formaldehyde (Merck Life Science AS), and stained with Giemsa (Merck Life Science AS). Colonies containing at least 50 cells were counted.

### *In vivo* xenograft study

All animal experiments were approved by the local ethical committee (approval ID: N231/14) appointed by the Swedish Board of Agriculture and conducted in accordance with the local guidelines and the European Directive 2010/63/EU.

Female, immunodeficient nude mice (NMRI-nu/nu, Taconic) were used for the xenograft studies. The animals were housed in cages containing 2–6 mice with ad libitum access to food and sterile water. The cages contained environmental enrichment (a house, nest material and gnawing sticks) and the mice were kept on a 12h day/night cycle.

Each mouse was anaesthetized (Isoflurane 4% induction and 2% maintenance) and injected subcutaneously (s.c.) on the right flank with 1×10^7^ SK-N-AS cells. After 24h, 11 animals were randomly selected for the pre-treatment group and received 10 mg/kg α-NETA s.c. daily. α-NETA was dissolved in 10% Captisol^®^ (Ligand Pharmaceuticals, Inc., La Jolla, USA). The mice were weighed and tumors were measured every other day. The tumor volume was calculated with the following formula: length × (width)^2^ × 0.44. When tumors reached a volume of ≥ 0.15ml the mice were randomized to either treatment group (20 mg/kg α-NETA, daily s.c. injections, n=11 in pre-treatment group and n=10 in treatment group) or control group (vehicle, daily s.c. injections, n=11). When the tumors from the pre-treatment group were ≥ 0.15ml the α-NETA dose was increased to 20 mg/kg.

The mice were closely monitored for weight loss and other signs of toxicity. In accordance with the ethical guidelines the animals were sacrificed when tumors reached a volume of 2ml, or a diameter over 2cm, and the tumors were resected. Hence, survival was defined as time needed for the animals to grow a macroscopic tumor (volume >1.5ml). Smaller parts of the tumors were fixed in formaldehyde or frozen.

Tumor volume growth was analyzed using rate-based comparison. By fitting each tumor's growth curve to an exponential model (by correlating the logarithm of the tumor volume measurements to the time), the slope, as an estimate for the tumor growth, for each tumor's growth could be determined [64].

### Statistics

SigmaPlot and GraphPad Prism software was used for the statistical analysis and the graphs. Differences between several groups were assessed with one-way ANOVA and Bonferroni post-test or two-way ANOVA and Dunnett's post-test. One sample t-test was used to compare differences between one group and a hypothetical mean. The survival analysis on tumor growth *in vivo* was performed using the Kaplan-Meier method and statistical differences between groups were performed using log-rank test.

## SUPPLEMENTARY MATERIALS FIGURES



## Abbreviations

CCRL2: C-C chemokine receptor-like 2
CMKLR1: chemokine-like receptor 1
GPR1: G-protein-coupled receptor 1
TME: tumor microenvironment
α-NETA: 2-(α-naphthoyl) ethyltrimethylammonium iodide
MMP: matrix metalloproteases
ICC: Immunocytochemistry
IHC: immunohistochemistry
IF-P: immunofluorescence on paraffin-embedded samples.
