# Supplementary material for: Inhibition of chemerin/CMKLR1 axis in neuroblastoma cells reduces clonogenicity and cell viability in vitro and impairs tumor growth in vivo
Source: Oncotarget. 2017 Jul 27;8(56):95135–51. doi: 10.18632/oncotarget.19619 (PMC5707011; doi:10.18632/oncotarget.19619)
Supplement: Supplementary file 1 [file oncotarget-08-95135-s001.pdf]

Inhibition of chemerin/CMKLR1 axis in neuroblastoma cells reduces clonogenicity and cell viability *in vitro* and impairs tumor growth *in vivo*

SUPPLMENTARY MATERIALS

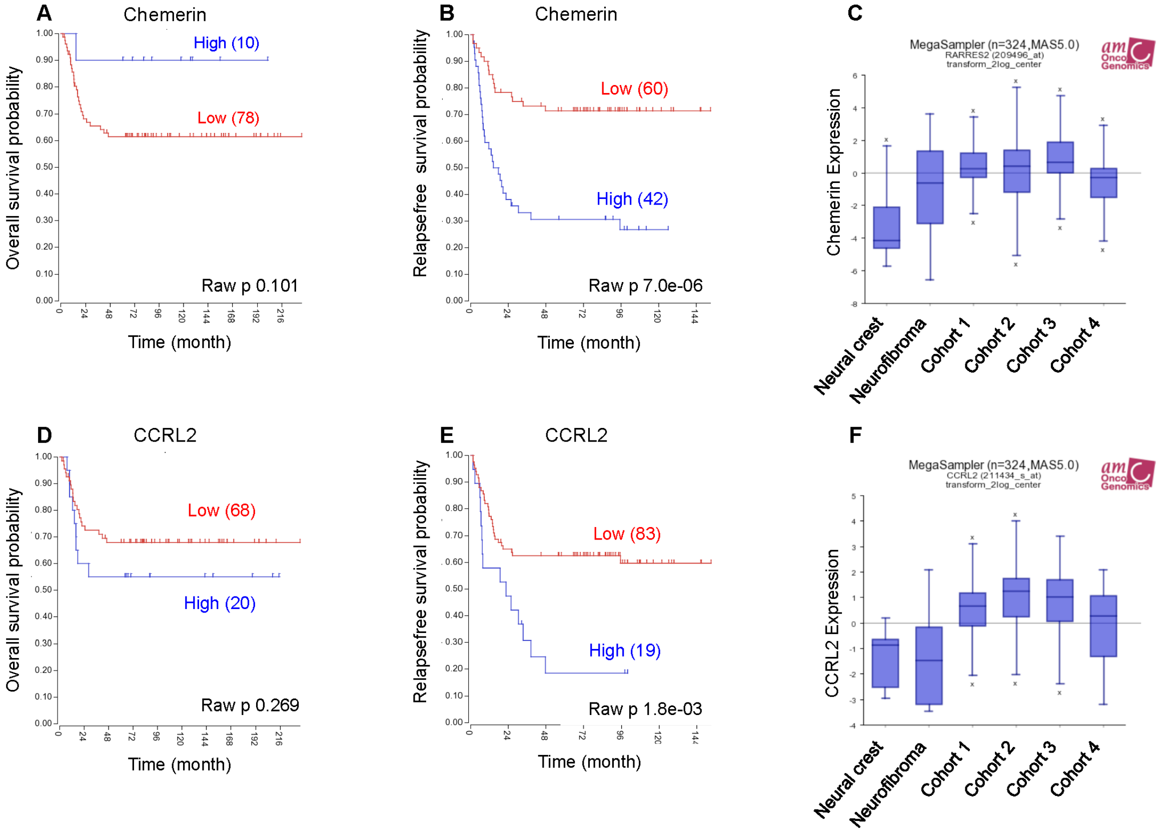

Supplementary Figure 1: Expression data was analyzed using the R2 database http://r2.amc.nl. Kaplan-Meier survival estimates were used to evaluate the prognostic value of *RARRES2* (chemerin) **(A, B)** and *CCRL2* **(D, E)** expression in two patient data sets (A and D: Versteeg n=88; B and E: Seeger n=102). The Kaplan-Meier scanning tool was used to determine the RARRES2 (chemerin) and CCRL2 mRNA expression in neuroblastoma. All expression data were scanned to find the most optimal cut-off between high and low gene expression and the log-rank test that gave the lowest p-value was calculated to search for significant differences between tumor samples expressing high and low RARRES2 (chemerin) and CCRL2 mRNA levels, respectively. The expression of *RARRES2* (chemerin) **(C)** and *CCRL2* **(F)** was compared between neural crest (Etchevers n=5), benign neurofibroma (Miller n=86) and 4 neuroblastoma cohorts (cohort 1: Versteeg n=88, cohort 2: Delattre n=64, cohort 3: Hiyama n=51, cohort 4: Lastowska n=30).

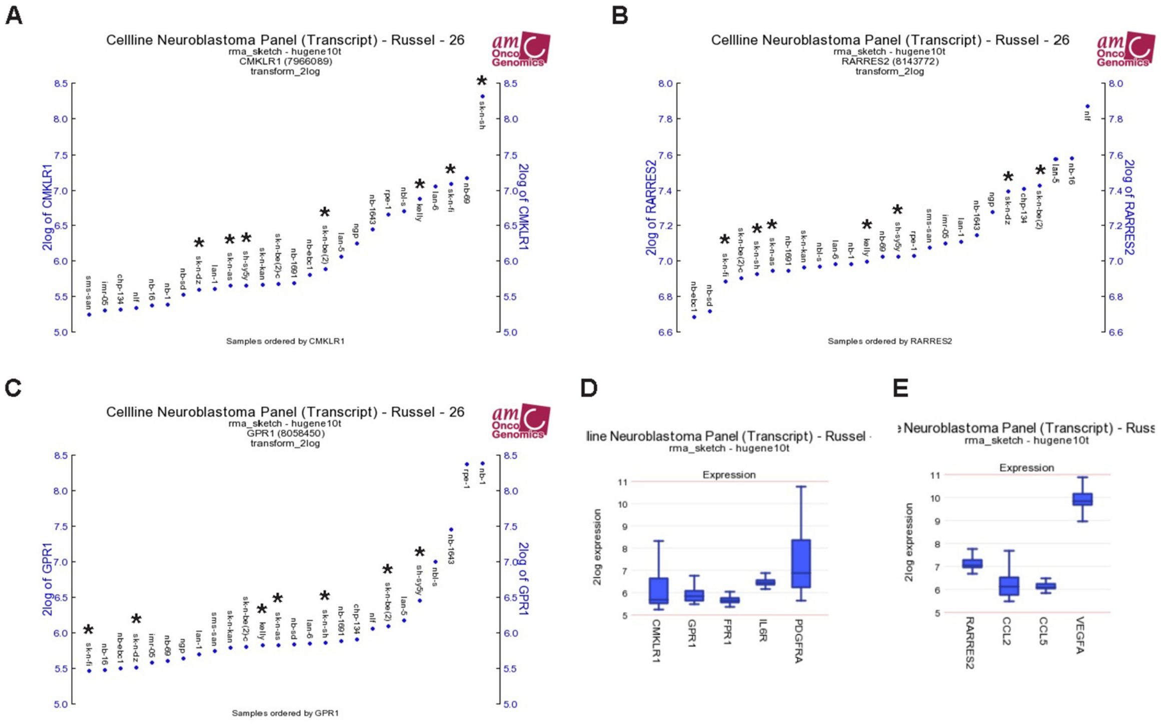

Supplementary Figure 2: *CMKLR1* **(A),** *RARRES2* **(B)** and *GPR1* **(C)** expression was analyzed using the Russel neuroblastoma cell line panel (transcript) in the R2 database http://r2.amc.nl. Cell lines used in this study were marked with an asterisk. The expression of *CMKLR1* and *GPR1* was compared to the previously in neuroblastoma cell lines described receptors *FPR1*, *IL6R* and *PDGFRA* **(D)**. Chemerin expression was compared to *CCL2*, *CCL5* and *VEGFA* **(E)**.

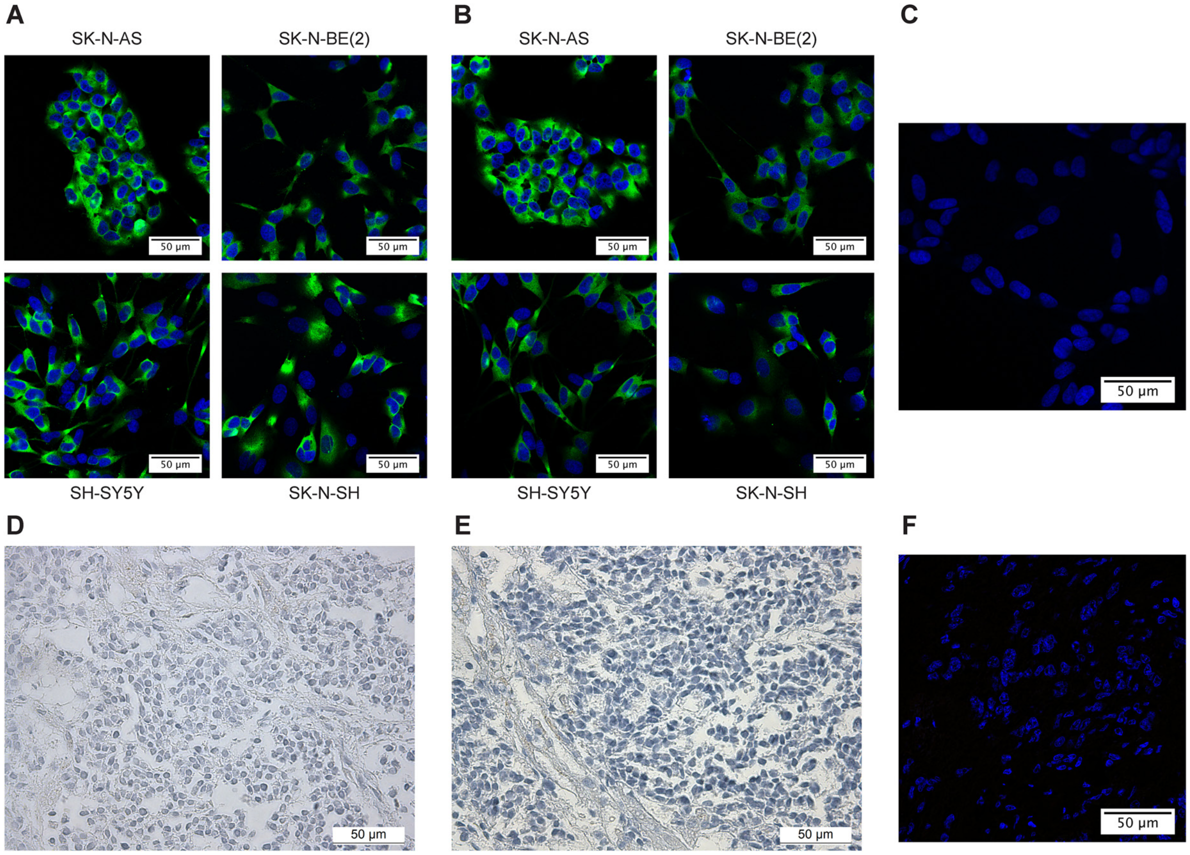

Supplementary Figure 3: Immunofluorescence CMKLR1 staining using SK-N-AS, SK-N-BE(2), SH-SY5Y and SK-N-SH cells and two different antibodies: STJ92262 from St. John's laboratory **(A)** and LS-B12924 from LSBio **(B)**. The used secondary antibody was Goat anti-Rabbit IgG (H+L), Alexa Fluor 488 (A-11008, Thermo Fisher Scientific) and the nuclei were stained with Hoechst 33342 (ImmunoChemistry Technologies). Isotype control staining for IF and IHC. **(C)** SH-SY5Y cells were incubated with rabbit isotype antibody instead of primary antibody. Immunoperoxidase labeled tissue sections where the primary antibodies were replaced with rabbit **(D)** or mouse **(E)** isotype antibodies. Neuroblastoma tissue sections were incubated with mouse and goat isotype antibodies prior to fluorescence staining **(F)**.

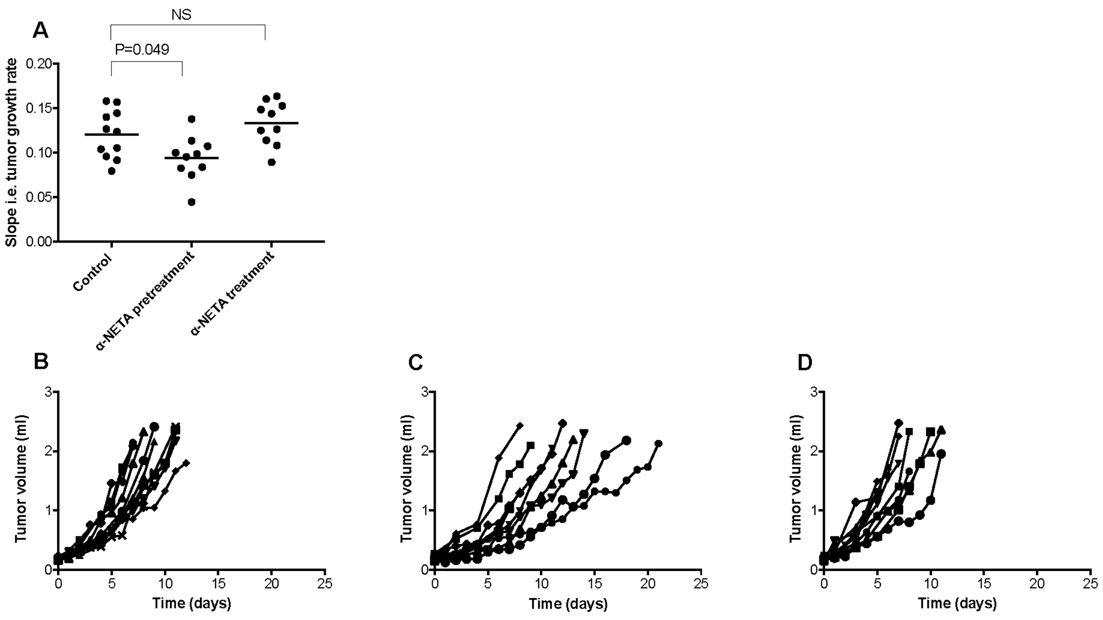

Supplementary Figure 4: The tumor growth rate in neuroblastoma bearing mice was significantly lower in the α-NETA pre-treatment group compared to the control group (one-way ANOVA P=0.0061, Bonferroni post-test, control vs. pre-treatment P=0.049). Growth rate in the different treatment groups was illustrated as the slope from curves over individual mice log tumor volume versus time. Goodness of curve fitting was assessed with R^2 (R^2 mean 0.97; range 0.875-0.999). The line represents mean slope in each treatment group **(A)**. Individual tumor growth in **(B)** control mice (daily s.c. injections with 10% Captisol®), **(C)** pre-treatment group (daily s.c. injections with 10 mg/kg α-NETA from the day after tumor cell injection and 20 mg/kg when the tumor reached 0.15ml) and **(D)** treatment group (daily s.c. injections with 20 mg/kg α-NETA after the tumor reached 0.15ml).

CMKLR1 vs. CD14, R=0.810, p=3.9e-59

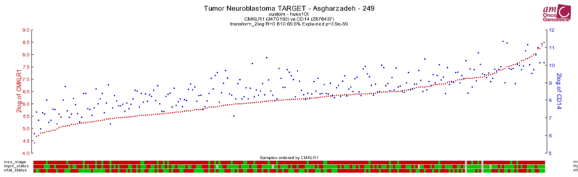

CMKLR1 vs. IL6R, R=0.632, p=3.2e-29

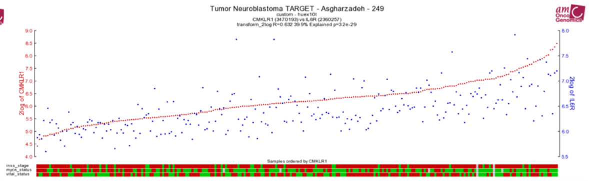

CMKLR1 vs. CD33, R=0.561, p=4.4e-22

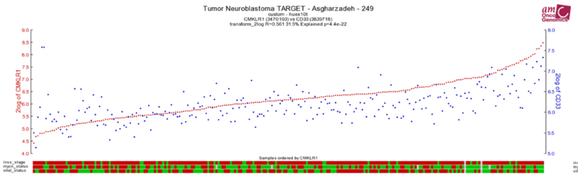

CMKLR1 vs. IL10, R=0.529, p=2.5e-19

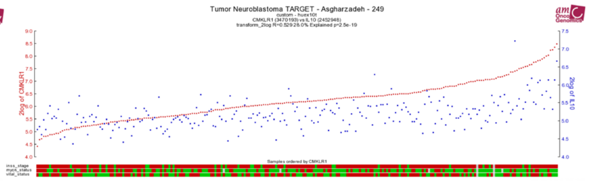

CMKLR1 vs. FCGR3/CD16, R=0.673, p=3.7e-34

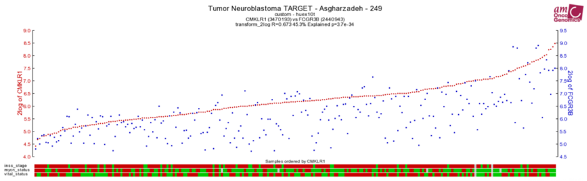

Supplementary Figure 5: Expression data was analyzed using the R2 database http://r2.amc.nl. The Asgharzadeh neuroblastoma cohort n=249 was used to correlate the expression of previously identified TAM markers (*CD33, CD14, IL6R, IL10, FCGR3*) to *CMKLR1* expression.

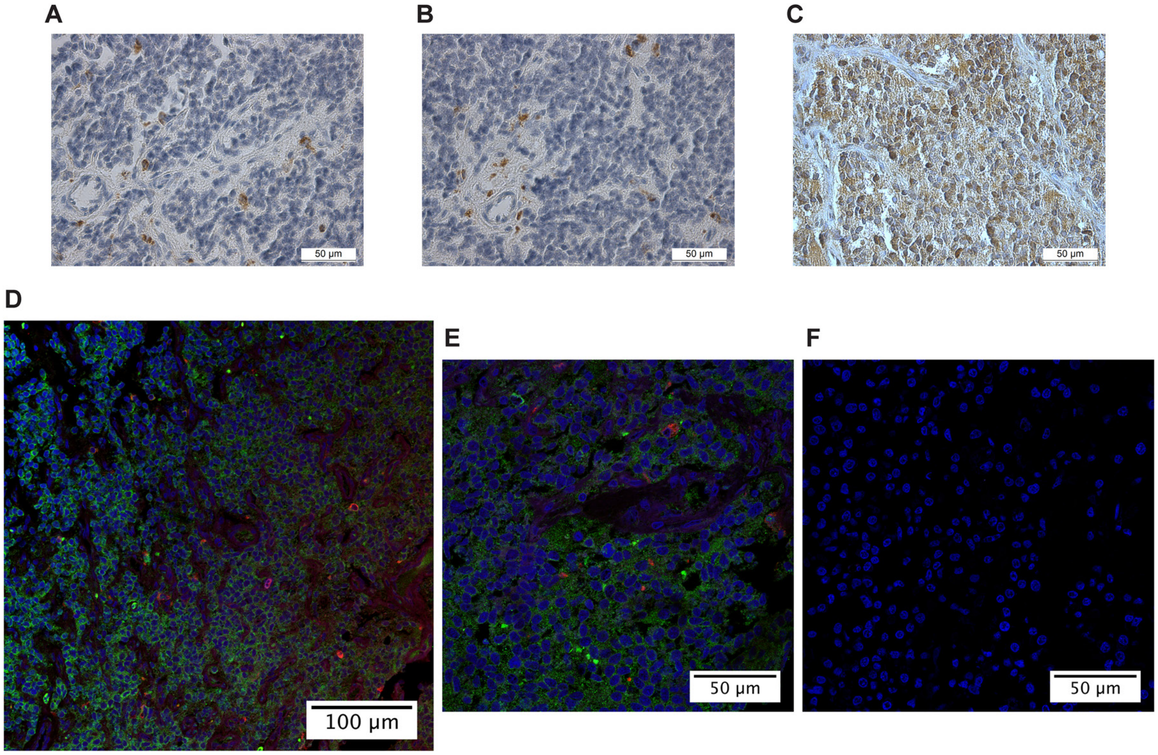

Supplementary Figure 6: Immunoperoxidase staining of the macrophage marker CD68 using M0814 from Dako **(A, B)** and CMKLR1 using STJ92262 from St. John's laboratory **(C)** in neuroblastoma primary tumor tissue demonstrates while the majority of cells in the TME are positive for CMKLR1 only few are positive for CD68. Double immunofluorescence labeling **(D, E)** of CMKLR1 (green) and CD68 (red) confirms this observation. The used secondary antibodies were Goat anti-Rabbit IgG (H+L), Alexa Fluor 488 (A-11008, Thermo Fisher Scientific) and Donkey anti-Mouse IgG (H+L), Alexa Fluor 594 (A-21203, Thermo Fisher Scientific). The nuclei were stained with DAPI (blue). Isotype control staining is displayed in panel **(F)**.

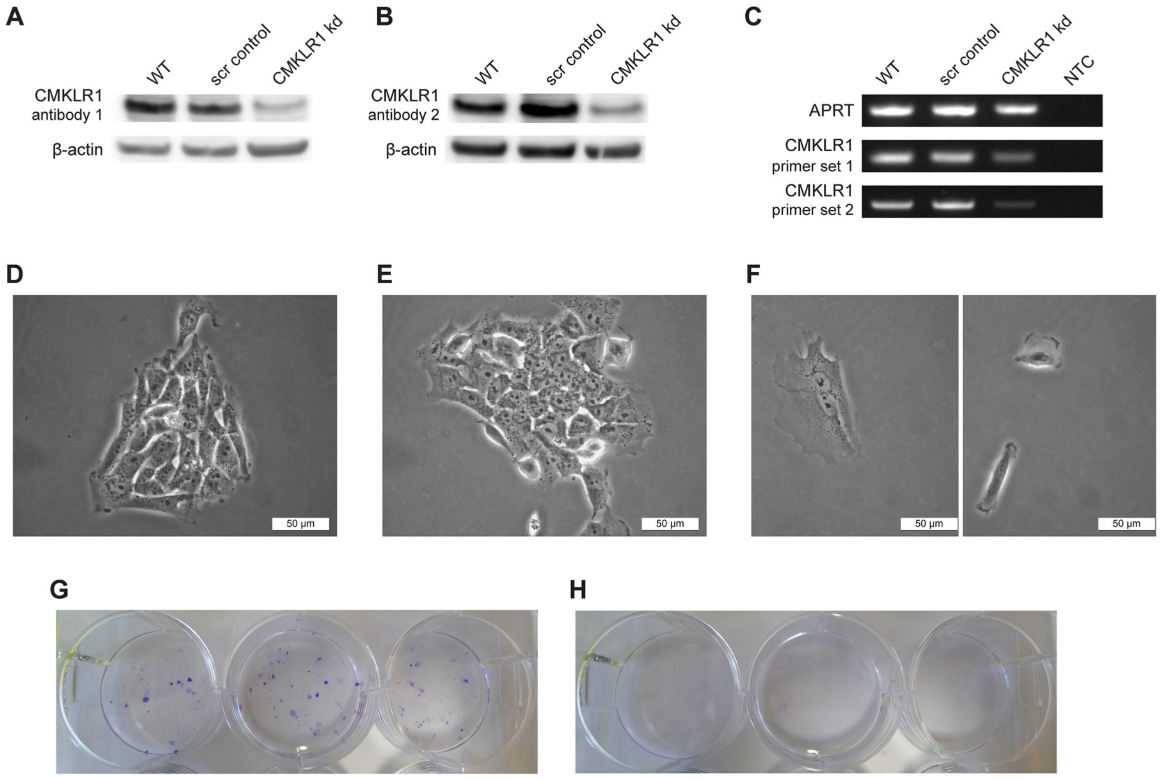

Supplementary Figure 7: SK-N-AS cells were transfected with sgRNA/Cas9 targeting all variants for human CMKLR1 or scrambled sgRNA control plasmids (GeneCopoeia, Inc., Rockville, USA) using jetPRIME® transfection reagent (Polyplus-transfection®, Illkirch, France) according to the provided manual. Forty-eight hours post transfection the cells were seeded as single cells into 96-well plates. After clonal expansion, the clones were screened for CMKLR1 expression using western blot and RT-PCR. Western blot of SK-N-AS wild-type (wt), scramble control (scr control) and CMKLR1 knockdown (CMKLR1 kd) using two different antibodies targeting CMKLR1 (**A**, STJ92262, St. John's laboratory and **(B)** ab64881, abcam) showing a clear reduction in CMKLR1 protein in the knockdown clone. RT-PCR analysis demonstrating a downregulation of CMKLR1 transcripts using two different primer sets **(C)**. In clonogenicity assays **(D-H)** slower growth and the formation of indistinct colonies was observed in CMKLR1 knockdown cells. While small but distinct colonies were observed 6 days after cell seeding in the wild-type **(D)** and scramble control **(E)** the CMKLR1 knock down cells multiplied slower and were only loosely connected **(F)**. After completion of the assays macroscopic colonies were observed in the scramble control **(G)** but not the CMKLR1 knockdown cells **(H)**.
